# Fitness and Fatness as Health Markers through the Lifespan: An Overview of Current Knowledge

**DOI:** 10.1097/pp9.0000000000000013

**Published:** 2018-04-02

**Authors:** Francisco B. Ortega, Cristina Cadenas-Sanchez, Duck-chul Lee, Jonatan R. Ruiz, Steven N. Blair, Xuemei Sui

**Affiliations:** a PROFITH “PROmoting FITness and Health through physical activity” research group, Department of Physical and Sports Education, Faculty of Sport Sciences, University of Granada, Spain; b Department of Kinesiology, Iowa State University, Ames, IA; c Department of Exercise Science, University of South Carolina, Columbia, SC.

**Keywords:** Physical fitness, cardiorespiratory fitness, fatness, obesity, health, preschool, children, adolescents, adults, older adults

## Abstract

There is an increasing body of evidence supporting that both fitness and fatness levels relate to current and future individuals’ health status. In this article, we discuss the meaning of fitness and fatness/obesity, and make an overview of what is currently known about fitness and fatness as potentially modifiable risk factors related to health and disease from preschool children to older adults. We describe the methods available for fitness assessment in each age group, providing reference/criterion values when available. Most of the existing previous reviews are focused on specific age groups with the advantage of allowing more in-depth analysis of the evidence, but the disadvantage of losing the overall understanding of the fitness and fatness binomial through the human lifespan, which is the ultimate goal of the present article.

## Introduction

**Fig 1.**
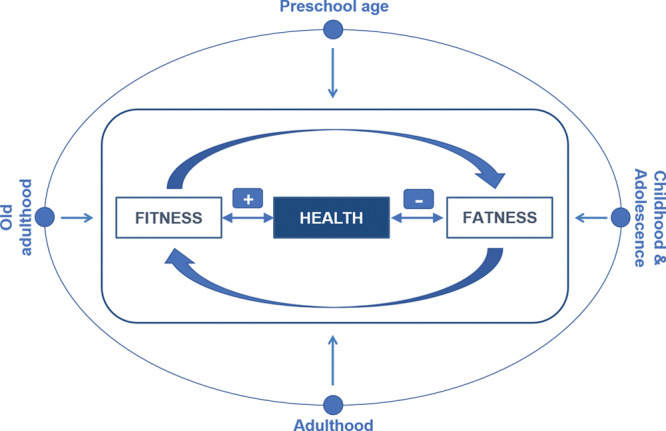
Graphical illustration of the key concepts and focus of the present article.

The physical fitness (hereinafter fitness) level of a person and the amount of adipose tissue accumulated in the body (hereinafter fatness) have both shown to have a strong link with many and diverse health outcomes.^[1–4]^ Although previous reviews have focused on specific age groups, we hereby aim to present in a single document an overview of the relevance for health of both fitness and fatness through the lifespan (Fig 1). In addition, we aim to briefly describe the methods available for fitness assessment in each age group, providing reference/criterion values when available. For this article, we have gathered a group of experts with long and extensive experience on fitness and fatness in some of the age groups studied, so that the writing group together can cover with proficiency this topic across the human lifespan.

## Definition of fitness

Physical activity, physical exercise, and fitness are related, but not interchangeable concepts. Physical activity is defined as any bodily movement produced by skeletal muscles that result in an increased energy expenditure. This broad term means that physical activity includes almost everything that a person does. Inactivity is defined as a level of activity considered insufficient, which in practical terms is defined as not meeting the International Physical Activity Guidelines for children or adults specifically (https://health.gov/paguidelines/). The concept of inactivity should not be mixed with sedentarism. The Sedentary Behaviour Research Network (http://www.sedentarybehaviour.org/sbrn-terminology-consensus-project/) defines sedentary behavior as any waking behavior characterized by an energy expenditure ≤ 1.5 MET while in a sitting or reclining posture. Based on these definitions, sedentarism and inactivity are different constructs and consequently a person can be at the same time active (if meeting the minimum physical activity guidelines of activity per week) and sedentary (if accumulating much time in sedentary behavior). Another related but not interchangeable term is physical exercise, defined as a subset of physical activity that is planned, structured, repetitive, and purposive to improve or maintain fitness.

Fitness is defined as a set of attributes related to a person’s ability to perform physical activities that require aerobic capacity, endurance, strength, or flexibility and is determined mostly by a combination of regular activity and genetically inherited ability. Although physical activity and sedentary time are behavior, fitness is an attribute.

Among the health-related physical fitness components, cardiorespiratory fitness (CRF) is the one that has been studied the most. CRF reflects the overall capacity of the cardiovascular and respiratory systems and the ability to carry out prolonged exercise. Many other terms have been used to refer to CRF: cardiovascular fitness, cardiorespiratory endurance, aerobic fitness, aerobic capacity, aerobic power, maximal aerobic power, aerobic work capacity, and physical work capacity. All refer to the same concept and are used interchangeably in the literature. Another important related concept is maximal oxygen consumption, or VO_2_max. The VO_2_max attained during a graded maximal exercise test (usually performed running, biking, or with step tests) is an objective measure of CRF level. Although different ways have been used to express VO_2_max, the most common way is as the volume of oxygen consumed per unit of time relative to body mass (ml/min/kg). However, researchers aiming to compare CRF level across groups of young people should consider the way in which the VO_2_max is expressed [ie, ml/min/kg of body mass or ml/min/kg of fat free mass (FFM) or l/min], because it can influence the results and interpretation, leading to misleading conclusions.

**Fig 2.**
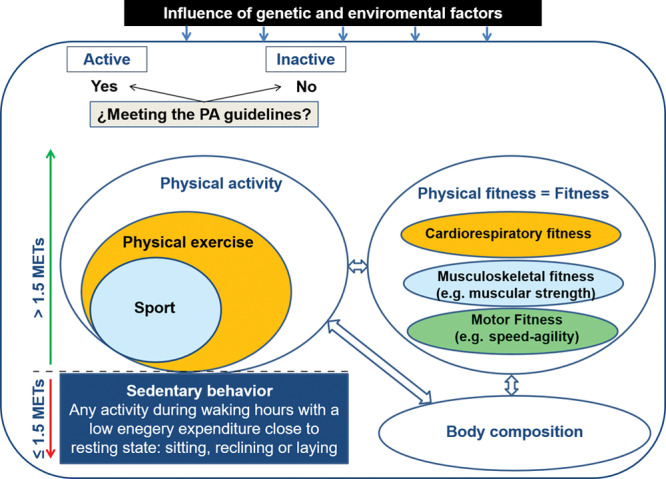
Graphical illustration of physical activity- and fitness-related concepts. PA = physical activity; METs = metabolic equivalents.

Other key health-related physical fitness components are muscular strength, speed, and agility. Muscular strength is the capacity to carry out work against a resistance. Because the maximum force that can be generated depends on several factors (eg, the size and number of muscles involved, the proportion of muscle fibers called into action, the coordination of the muscle groups, neuromuscular function, etc.), there is no single test for measuring complete muscle strength. The main muscular strength components are maximal strength (isometric and dynamic), explosive strength, endurance strength, and isokinetic strength. Speed refers to the ability to perform a movement within a short period of time, and agility is the ability to rapidly change the position of the entire body in space with speed and accuracy (Fig 2).

## Definition of fatness/obesity

**Fig 3.**
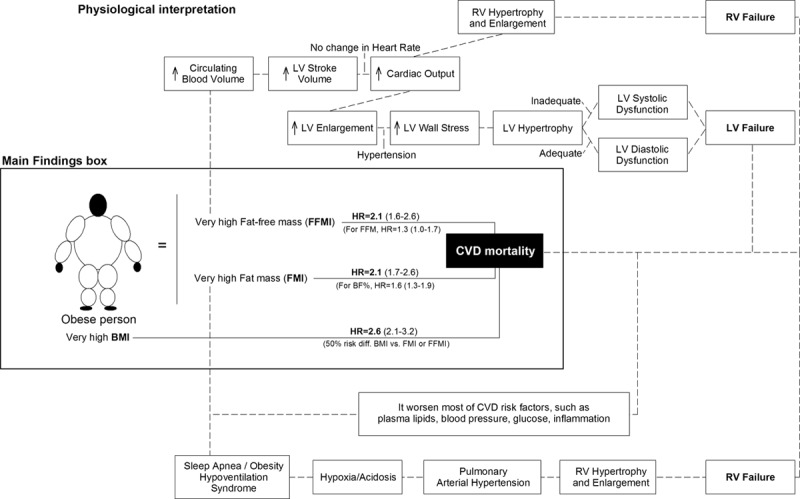
Graphical illustration of the associations of BMI, BF%, FMI, and FFMI with CVD mortality and physiological explanation. Reproduced with permission from Ortega et al.^[5]^ BMI = body mass index, BF% = Body fat percent, CVD = cardiovascular disease; HR = Hazard Ratios; LV = left ventricular; RV = right ventricular.

Body fatness and obesity are most often used in the literature as synonymous, and they refer to the state of having excess adipose tissue in the body, which is related to poorer health. However, although obesity is generally understood as an excess of body fat, it is internationally and well-acceptedly defined as a high body mass index (BMI), that is, equal or higher than 30 kg/m^2^. There is therefore a mismatch between the concept (an excess of fat) and the most widely used index to measure and define obesity, a high BMI, indicating an excess of body weight normalized by height. Probably, the reasons for the more frequent use of BMI than body fat indices to define obesity include that the measure of BMI is simpler, requires less expensive equipment, and training than the measure of body fat indices. There is however a general belief that BMI is a proxy of the amount of adiposity and that whenever possible, a more accurate metric of body fat, such as body fat percent (BF%) or fat mass index (FMI, fat mass expressed in kilogram divided by height squared expressed in meters) should be used to define obesity and to study the relationship between obesity and health. However, most of currently available evidence supporting the negative consequences of obesity on health outcomes is based on BMI-defined obesity. Therefore, someone would wonder whether obesity could be defined as an excess of body weight, rather than an excess of body fat as usually stated. Using data from the Aerobics Center Longitudinal Study (ACLS), we tested the hypothesis of whether an accurate measure of BF% and FMI as assessed by hydrostatic weighing (considered a gold standard method for body composition) in more than 30,000 participants would be a stronger predictor of cardiovascular disease (CVD) mortality than the simple and inexpensive BMI.^[5]^ Unexpectedly, having a very high BMI was a significantly stronger predictor than both a very high BF% and FMI of CVD mortality. These findings suggest that despite BMI is a poor index of body composition, because it does not discriminate between fat mass and FFM, it is perhaps a very good index of future health/disease, and particularly for CVD. The explanation for this novel finding seems to be related to the fact that a very high FFM index (FFMI, FFM expressed in kilogram divided by height squared expressed in meters) was also positively associated to higher risk of CVD death. Consequently, BMI, which is the mathematical sum of FMI + FFMI (both positively associated with CVD mortality) was a stronger predictor of CVD mortality than both FMI and FFMI separately. In that study, we provided a physiological explanation for why both a high FMI and a high FFMI are related to worse CVD prognosis (Fig 3). See the original article published by Ortega et al.^[5]^ for further information. These findings are not opposed to the notion that promoting FFM and particularly avoiding a low FFM is beneficial for health, because it increases the metabolism and prevent obesity and certain metabolic-related disorders, which our data show is that a very high (extreme) amount of FFM (which in our study set was defined as 80 kg of FFM and over) might be an extra burden for the heart for the mechanisms explains in Fig 3 and potentially increases the risk of CVD. These findings also question what the real meaning of obesity is: an excess of body fat (ie, high BF% or FMI) or an excess of body weight for a given body height (ie, high BMI)? The results hereby presented support the definition of obesity as an excess of body weight, given the stronger association with CVD of BMI compared with BF% or FMI, but there is a need of future studies to confirm or contrast these findings.

## Fitness and fatness in preschoolers

Fitness is a powerful marker of health in children and adolescents,^[6]^ and there is no reason to believe that fitness is less important in preschoolers. We systematically reviewed the existing literature focused on the validity, reliability, and the relationship with health of fitness tests used in preschool children. This systematic review concluded with a proposal of fitness test battery for preschoolers (aged 3–5 years) called the PREFIT (assessing FITness in PREschoolers) battery.^[7]^ On the contrary than in other age groups, CRF was the less studied fitness component, and balance the most.

**Fig 4.**
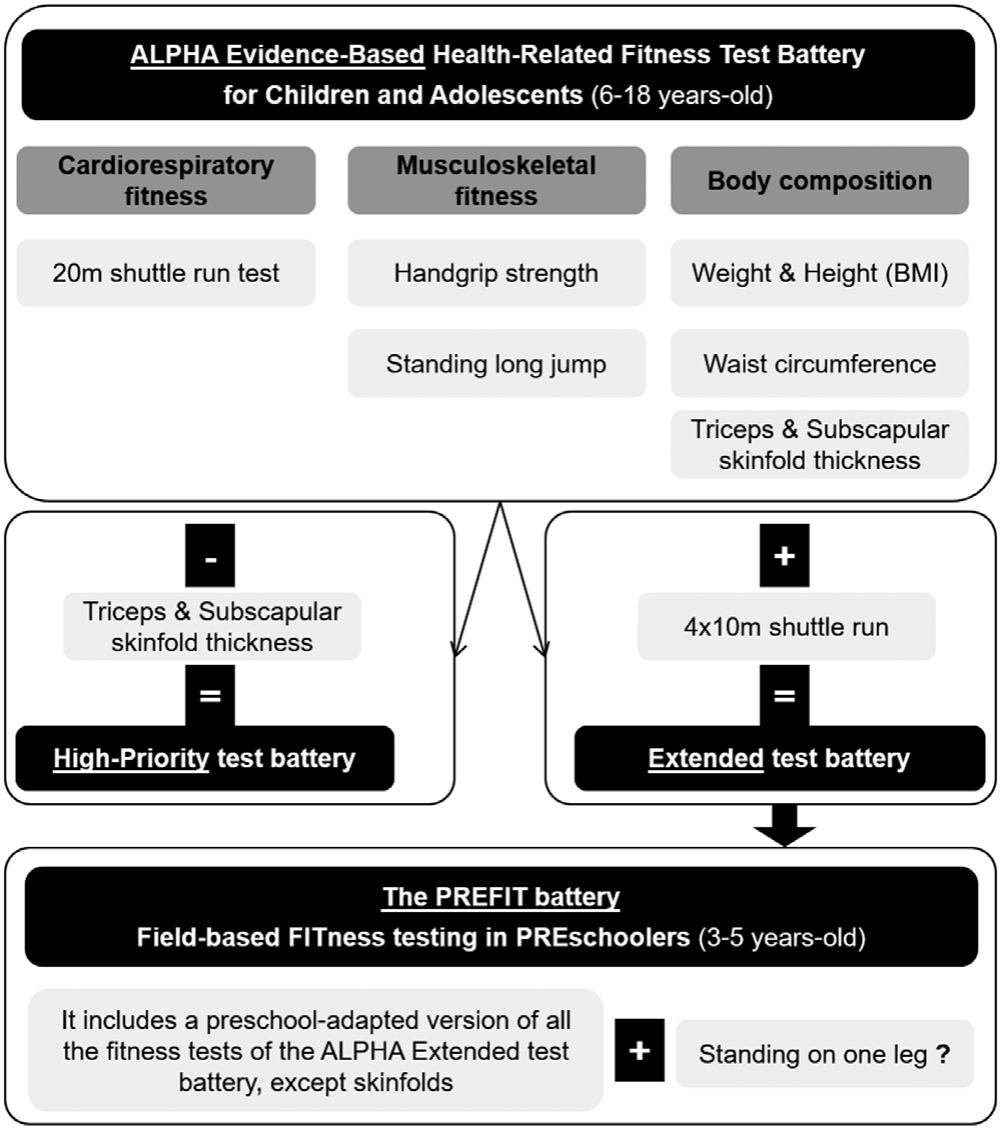
Evidence-based fitness test batteries to assess fitness in preschoolers, children, and adolescents. Adapted from Ruiz et al.^[12]^ and Ortega et al.^[7]^ with permission of the publisher. It is important to note that the fitness tests included in the PREFIT battery have major adaptations so that can be used in preschool children. As an example, the 20 m shuttle run test initial speed was reduced from 8.5 to 6.5 km/h, because the original speed was too difficult for 3–5 year olds. It is important to note that the standing on 1 leg test was selected based on the systematic review, but later testing in preschool children has shown poor reliability, being therefore its usefulness in this age group highly questionable (this is represented in the figure with the symbol “?”). Detailed information on the adaptations, practical consideration, and reliability of the tests included in the PREFIT battery has been published elsewhere.^[8]^ The standing on 1 leg test has shown a poor reliability in this population, being therefore highly questionable its usefulness in this age group. ALPHA = Assessing Levels of PHysical Activity and fitness; BMI = body mass index; PREFIT = assessing FITness in PREschoolers.

The PREFIT battery is composed of the PREFIT 20m shuttle run test (CRF), handgrip strength test (upper-limbs muscular strength), standing long jump (lower-limbs muscular strength), 4 x 10 m shuttle run test (speed-agility) and the standing on 1 leg test (balance; Fig 4 in the next section). It is important to note that the standing on 1 leg test was selected based on the systematic review, but later testing in preschool children has shown poor reliability, being therefore its usefulness in this age group highly questionable. Detailed information on the reliability of these tests in preschool children, and practical information on how to successfully conduct the tests at this early stage in life is published elsewhere.^[8]^ Manual of operation, explanatory videos and articles related to the PREFIT fitness test battery are available at http://profith.ugr.es/prefit?lang=en and http://profith.ugr.es/recursos-prefit?lang=en.

Concerning fatness, a growing body of literature has shown that childhood obesity has become a major health problem in children under 5 years of age, achieving epidemic proportions and reaching higher levels of overweight/obesity prevalence (including an important percentage in morbid obesity or obesity type III). The most recent (June 2017) data about the prevalence of obesity in preschool children aged 3–5 years show that 7–8% of children of this age are obese worldwide.^[9]^ Obesity in children has been related to many cardiovascular or metabolic problems among others. We have provided sex- and age- anthropometric and physical fitness reference standards based on a multicentre project with more than 3,000 preschoolers from 10 different regions geographically distributed across Spain (manuscript under review). An automatic calculator has been developed to compare the fitness or fatness level of preschool children, both at individual and at (large) group level. This calculator will available soon at http://profith.ugr.es/recursos-prefit?lang=en.

### Summary and future research directions

The PREFIT project (systematic review plus methodological articles and reference values) has contributed to a better understanding on the importance on why and how to assess fitness in preschool children aged 3–5 years. Future studies have the opportunity to evaluate the effectiveness of lifestyle intervention at early stages in life (eg, reducing sedentary behaviors, increasing active playing, and commuting, etc.) on improving fitness. Also, cohort studies can be designed and conducted from preschool ages, providing valuable information about how fitness track from early to older ages in life, and exploring which factors modulate this development. Future studies will be able to determine the capacity of fitness at preschool age to predict future diseases.

## Fitness and fatness in children and adolescents

Whereas in preschoolers CRF is the least studied fitness component, in children and adolescents CRF is by far the most studied fitness component in relation with health outcomes. Nowadays, there is little doubt about the association between poor CRF and CVD risk factors in children and adolescents.^[6]^ Under the umbrella of the European-funded Assessing Levels of PHysical Activity and fitness (ALPHA) project, we conducted a systematic review of 42 longitudinal studies that assessed the relationship between CRF levels in youth and future health and concluded that there was strong evidence suggesting that higher levels of CRF in childhood predicts a healthy CVD risk profile (ie, lower levels of blood lipids, blood pressure, and overall and central adiposity) in adulthood.^[10]^ There was moderate evidence supporting CRF levels in childhood as a predictor of future metabolic syndrome and arterial stiffness. Lastly, moderate evidence supported increases in CRF being inversely associated with changes in blood lipids and lipoproteins in adulthood.^[10]^ Furthermore, these results are supported by a series of articles from the Swedish Registries involving more than 1 million male adolescents followed up for roughly 35 years (see the study of Crump et al.^[11]^ as an example of them). This unique dataset has consistently shown that a low CRF level in adolescence is a strong predictor of different CVD-related outcomes in adulthood, such as hypertension, type-2 diabetes, stroke, ischemic heart disease, and overall mortality. Collectively, these findings support the notion that CRF levels among children and youth are not only an indicator of current health but also an indicator of future disease risks.

CRF has historically been included in almost all children and adolescents’ fitness test batteries.^[12]^ CRF test scores were originally tracked as a marker of sport performance, but the increasing focus on health-related fitness has led to its use as a screening test to identify children and adolescents at increased risk of future diseases. Although fitness testing has been a standard procedure in school settings as part of the physical education classes, the assessment of fitness in young people as a health marker in clinical or health settings is not a standard practice yet, despite the fact that health-related fitness standards already exist and allow meaningful interpretation of fitness assessment. In this context, we recently performed a systematic review and meta-analysis of the relationship between poor CRF and CVD risk factors that included 9,280 children and adolescents aged 8–19 years, from 14 countries.^[13]^ We observed that boys with low CRF (< 41.8 ml/kg/min) had a 5.7 times greater likelihood of having CVD risk [95% confidence interval (CI), 4.8–6.7). The comparable diagnostic odds ratio for girls with low CRF (< 34.6 ml/kg/min) was 3.6 (95% CI, 3.0–4.3). Therefore, CRF levels below 42 and 35 ml/kg/min for boys and girls, respectively, should raise concerns. These cut points identify children and adolescents who may benefit from primary and secondary cardiovascular prevention programming. In addition to the well-established relationship between CRF and physical health, there is emerging evidence suggesting that fitter kids have also better mental health, healthier brains, and better cognitive/academic performance.^[14,15]^ This is an exciting new field that is developing fast in parallel with the better understanding of the brain.

Although CRF has been by far the most studied and main health-related fitness component, emerging evidence suggests that a low muscular strength level is also a risk factor for many physical and mental diseases. In this context, we observed in more than 1 million male adolescents that those with a very low muscular strength level (ie, those in the first decile) as assessed by handgrip strength and knee extension strength tests had a significantly higher risk of premature all-cause mortality, and mortality due to CVD.^[16]^ In addition, we observed that a low muscular strength was related not only to worse physical health in the future but also to a significantly increased risk of having a psychiatrist-diagnosed mental disease in the future and with a higher risk of dying due to suicide. In this line, Smith et al.^[17]^ published a systematic review and meta-analysis focused on the physical and psychological health benefits of having a good muscular strength in childhood and adolescence, including both observational and intervention studies. They found strong evidence of an inverse association of muscular strength with total/central adiposity, and metabolic and CVD risk factors. Likewise, they found strong evidence of muscular strength to be positively associated with bone health and with self-esteem.

Given the relevance for health of CRF and muscular strength at these ages, the European Union funded the ALPHA project to provide standard protocols for assessing fitness at population levels for monitoring purposes. Although, treadmill and bike incremental laboratory tests are and will be the gold standard methods, they are often not feasible for assessment of large amount of people, situations in which field-based fitness tests are useful and valuable. Under the umbrella of the ALPHA project, we conducted a narrative review, 3 systematic review, and a number of methodological articles to end up proposing the evidence-based ALPHA fitness test battery for children and adolescents.^[12]^ Fig 4 shows the tests with the highest validity, reliability, and relation with future health and that are therefore proposed to be used for fitness testing in children and adolescents. One year later, the Institute of Medicine (United States), after thorough review of the literature, came to the same conclusion and proposal of tests to be used in this age group, providing therefore intercontinental (Europe-America) agreement on which fitness test should be used in children and adolescents. More information about the fitness protocols, videos, and reference values are available at http://profith.ugr.es/alpha-children?lang=en.

Finally, having obesity at childhood or adolescence is associated with a myriad of negative consequences already at these ages, but also later in life. The latest estimates indicate that around 5% of the children and adolescents are currently obese worldwide.^[9]^ It is worrisome that although the prevalence of obesity among children is lower than in adults, the rate of increase since 1980 to date in childhood obesity in many countries has been greater than the rate of increase in adult obesity, suggesting the need of actions to tackle obesity in early stages in life. Several prospective cohort studies have linked a high BMI in childhood and adolescence with higher risk of CVD and all-cause mortality in adulthood. In addition, it has been shown that CVD risk factors linearly impair as the degree of the obesity increases in children and adolescents,^[18]^ clearly indicating the adverse consequences of being severe and morbid obese early in life. There is emerging evidence suggesting that in addition to targeting a reduction in body weight and fat in obese youth, improving CRF could, to some extent, counteract the negative consequences of childhood obesity, the so-called Fat but Fit paradox.^[19]^ These findings support a public health message, both fatness/weight reduction and fitness improvements should be promoted in parallel in obese children and adolescents.

### Summary and future research directions

Existing cross-sectional and longitudinal studies have clearly and consistently shown that a better CRF and muscular strength in childhood and adolescence is related to better physical health, mental health, and brain/cognition. Future intervention studies should step further and formally test by means of mediation analyses whether exercise-induced improvements in fitness lead to improvements in these health outcomes. Likewise, the potential of fitness to attenuate the negative consequences of obesity in relation with different health outcomes needs to be further investigated.

## Fitness and fatness in adults

As in children and adolescents, in adults, CRF has been the most studied fitness component by far. Back in the late 80’s, we published data from the ACLS on fitness and fatness in relation to all-cause mortality and CVD mortality. Later ACLS studies showed that moderate-to-high CRF was associated with a lower risk of CVD mortality (compared with low CRF) in smokers and nonsmokers, in those with and without elevated cholesterol levels or elevated blood pressure, and in healthy and unhealthy individuals. ACLS reports also demonstrated that regardless of the initial CRF level, those individuals who maintained or improved their CRF level over a 5-year follow-up period also had a marked reduction in CVD and all-cause mortality. From these early studies to date, an enormous number of additional studies have consistently confirmed that CRF is a powerful marker of cardiovascular health at any age, sex, or health/disease condition, in both normal weight and obese individuals.^[20]^ In this context, the American Heart Association has recently published a Scientific Statement entitled “Importance of Assessing CRF in Clinical Practice: A Case for Fitness as a Clinical Vital Sign.”^[21]^ The group of experts coauthoring this statement conclude that there is consistent evidence, which indicates that assessing CRF significantly improves CVD risk classification algorithms and, therefore, patient management. Consequently, CRF assessment in clinical settings is strongly encouraged, and the authors provide some alternatives in cases in which maximal or submaximal incremental exercise texting are not feasible. It is also important to highlight a major advancement in this field, the recent creation of the Fitness Registry and the Importance of Exercise: A National Data Base.^[22]^ To our knowledge, these are the most accurate, largest, and most updated fitness reference values covering all the adult age groups to date. This data registry is based on 7,783 maximal (respiratory exchange ratio ≥ 1.0) treadmill tests conducted between 2014 and 2015 from men and women (aged 20–79 years) without CVD. Equivalent reference values derived from maximal bike tests, also from Fitness Registry and the Importance of Exercise: A National Data Base, are currently available (N = 4494). These reference values allow proper interpretation of CRF assessment for each specific age and sex, making CRF assessment more useful and meaningful.

**Fig 5.**
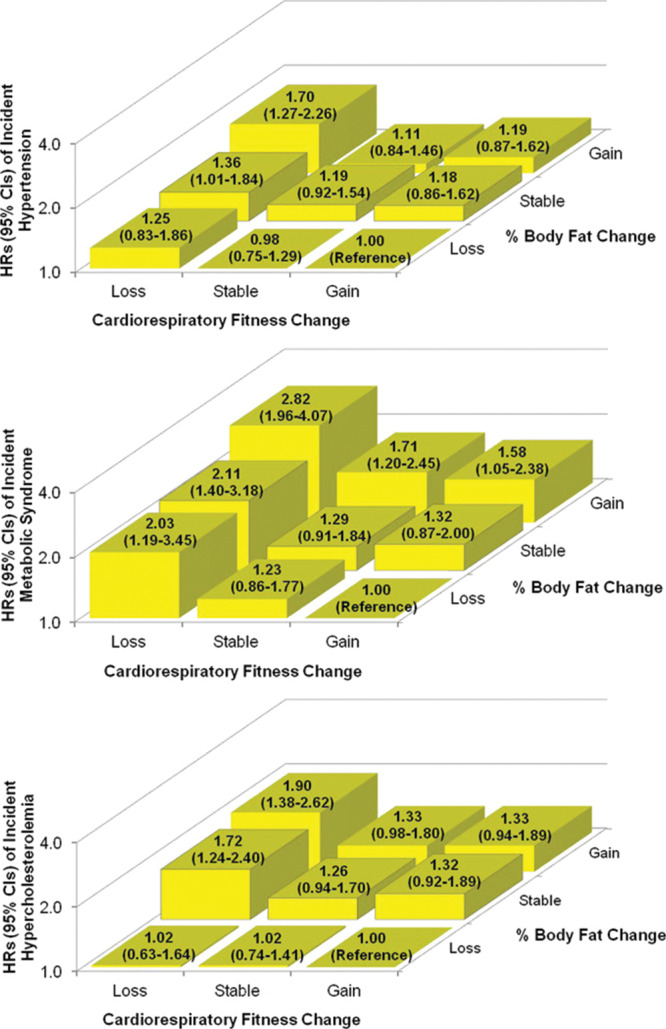
Hazard ratios (95% CI) of incident CVD risk factors by combined categories of changes in CRF and percentage body fat. Reproduced with permission from Lee et al.^[23]^ CI = confidence interval.

In adults, fitness and fatness are so tightly connected that it is difficult to understand one without the other. In this context, Dr. Lee et al.^[23]^ have made major contributions to better understand how fitness and fatness counteract to each other longitudinally in relation to future disease. In 1 of these studies using the ACLS data, the authors examined the influence of changes in fitness and fatness in the development of CVD risk factors, particularly hypertension, metabolic syndrome, and hypercholesterolemia.^[23]^ They observed that the increased risks associated with fat gain appeared to be attenuated, although not completely eliminated, when fitness was maintained or improved. In addition, the increased risks associated with fitness loss were also somewhat attenuated when fatness was reduced (Fig 5). Therefore, the authors concluded that both maintaining or improving fitness and preventing fat gain are important to reduce the risk of developing CVD risk factors in healthy adults. In a similar study also from the ACLS, the authors explored the hypothesis of whether fitness and fatness also counteracted in relation to all-cause and CVD mortality.^[24]^ In this case, maintaining or improving fitness was consistently associated with a lower risk of all-cause and CVD mortality; however, changes in BMI or BF% were not associated with of all-cause and CVD mortality, suggesting better health benefits with fitness improvements than with weight/fat reductions.

**Fig 6.**
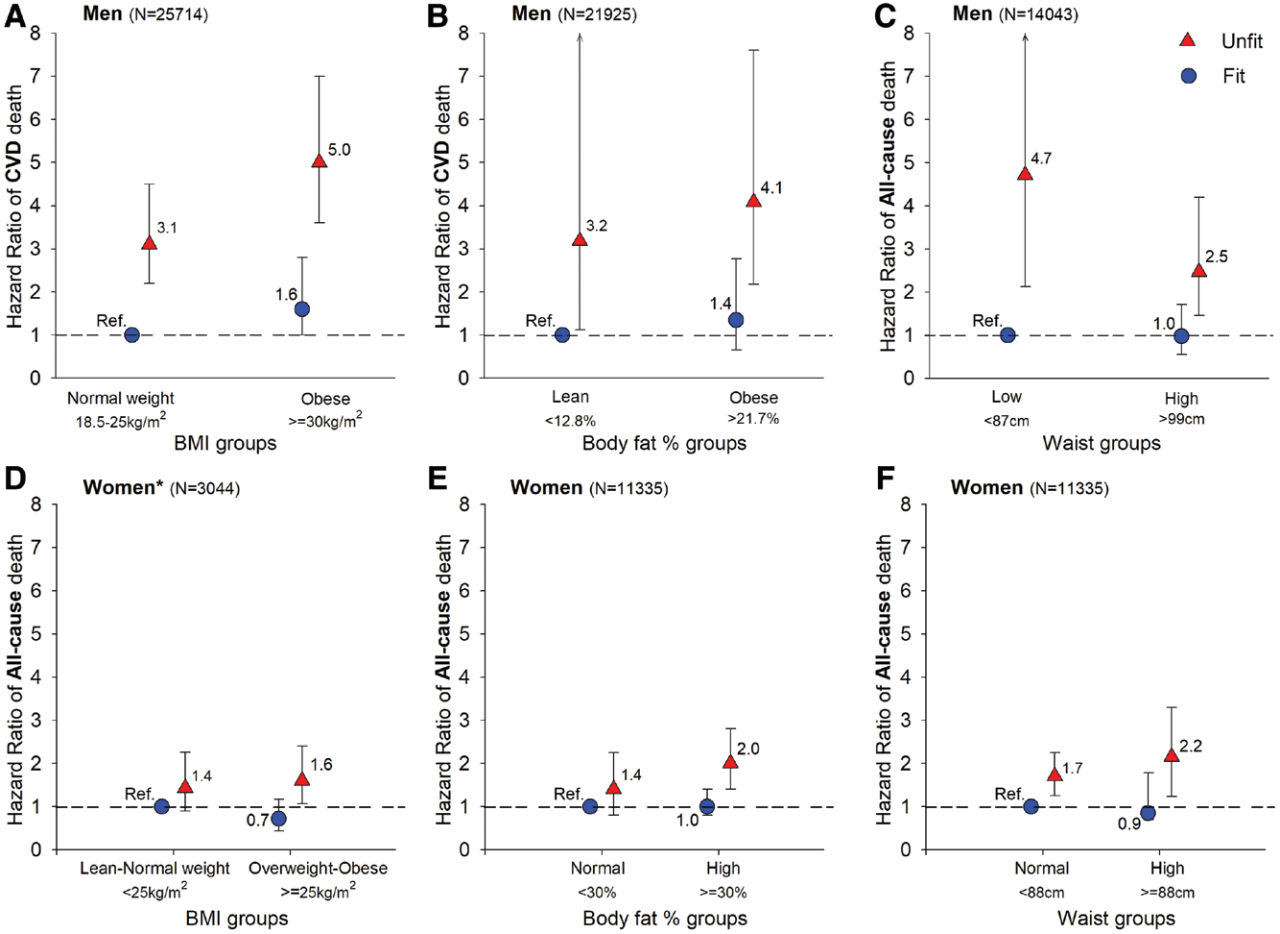
Illustration of the Fat but Fit paradox in relation with CVD mortality and all-cause mortality in men and women. Reproduced from Ortega et al.,^[19,25]^ with permission of the publishers. Unfit-fit was categorized as below-above the age- and sex-specific percentile 20th within each original study. BMI = body mass index; CVD = cardiovascular disease.

In line with these findings, a number of studies have shown that a moderate-to-high CRF largely attenuates the negative effects of being obese, and that the evidence of this is especially strong for CVD.^[1,25]^ This phenomenon is known as the Fat but Fit paradox, which is mainly based on 2 major findings observed in several studies: (1) obese but fit individuals (ie, moderate-to-high CRF), have marked reduction in risk compared with their obese unfit counterparts; and (2) keeping your weight within the normal range might not be enough, there are data showing that a normal-weight person but unfit, can have a similar or even higher risk of CVD mortality than an obese but fit person (Fig 6).^[19]^

In addition to the strong link between CRF and health, emerging evidence supports that muscular strength is also related to better health, in isolation and in conjunction with fatness. In this part of the review, we would like to address the following 2 specific research questions. First, is muscular strength or body fatness more important, specifically regarding mortality in adults? Second, can high muscular strength reduce, if not completely eliminate, the negative effects of body fatness on mortality? The answers to these questions will promote the development of more efficient and effective public health strategies, particularly because reducing body fatness is more complicated and usually challenging than increasing muscular strength by resistance exercise in general populations.

Handgrip strength is a widely used test for muscular strength because it is simple, easy to use, and well correlated with health outcomes including mortality.^[26]^ A recent analysis of over 400,000 adults aged 40–69 years from the U.K. Biobank study found that muscular strength is more important than body fatness.^[27]^ Specifically, results indicated that the weakest men (lowest fifth) in the least fat group (% body fat ≤ 20%) showed 89% [hazard ratio (HR), 1.89; 95% CI, 1.52–2.36] increased risk of all-cause mortality, while the strongest men (highest fifth) in the most fat group (% body fat > 25%) showed no increased risk (HR, 1.02; 95% CI, 0.84–1.25), compared with the strongest and least fat men. They also found that higher muscular strength almost eliminated the negative effects of excess fatness on mortality. The weakest men in the most fat group had 39% increased risk of death (HR, 1.39; 95% CI, 1.14–1.68), but this risk was negligible and no longer statistically significant (HR, 1.02; 95% CI, 0.84–1.25), among the strongest men in the same most fat group. The authors also observed mortality benefits of higher muscular strength in other fatness groups. They observed similar results in women, for CVD mortality, and when BMI and waist circumference were used as measures of fatness, although the negative effects of fatness on mortality were not completely eliminated in some analyses.

Another method of assessing whole body muscular strength is the 1-RM (repetition maximum) test, which is typically performed using bench and/or leg press machines. A study using the ACLS data of over 8,700 men aged 20–80 years found that both muscular strength (based on 1-RM bench and leg presses) and body fatness (based on BMI) were equally important on all-cause mortality.^[28]^ Specifically, the age-adjusted all-cause mortality rate was similar between the weakest men (lower third) with normal weight (33 per 10,000 person-years) and strongest men (upper third) with overweight/obesity (34 per 10,000 person-years), but lower (21 per 10,000 person-years) in the strongest, normal weight men. However, higher muscular strength reduced the negative effects of overweight/obesity on mortality. Specifically, the weakest men in the obesity group had the highest death rate (42 per 10,000 person-years), but this increased death rate was reduced in men with moderate (middle third) (26 per 10,000 person-years) or high (upper third) (34 per 10,000 person-years) muscular strength level in the same obesity group. Similar results were indicated in cancer mortality. The authors found mortality benefits of higher muscular strength in both normal and overweight/obese men.

Given the relevance for health of CRF and muscular strength in adults, fitness assessment is strongly recommended from a public health and clinical point of view. In addition to the treadmill and bike tests conducted in the laboratories, there are field-based alternatives that are cheaper and in many situations more feasible. In this context, under the ALPHA project an evidence-based fitness test battery was proposed to be used in adults and protocols, videos and reference values are available elsewhere: http://www.ukkinstituutti.fi/en/alpha.

### Summary and future research directions

There is consistent evidence supporting that a moderately to high CRF in adulthood and improvements in CRF are associated with lower risk of a myriad of health outcomes later in life. Fitness and fatness seem to counteract with each other particularly in the association with CVD mortality, with fitness largely attenuating the adverse consequences of obesity. Yet much less evidence than for CRF is available, a moderate-to-high muscular strength level has also shown to be related to a lower risk of future diseases and seems also to attenuate the negative effect of obesity in certain health outcomes. Collectively, we can conclude that current evidence supports the implementation of exercise programs aiming to improve both CRF and muscular strength in any adult of any condition, and as a parallel goal to the body fat/weight reduction in obese adults. There are nowadays standard protocols for CRF assessments and reference values available to correctly interpret such assessment. In addition, future long-term randomized controlled trials including different study groups aiming CRF improvement alone, muscular strength alone, and a combination of both, will be able to definitively determine the cause-effect of improving these 2 main fitness components on a selected number of health outcomes.

## Fitness and fatness in older adults

**Fig 7.**
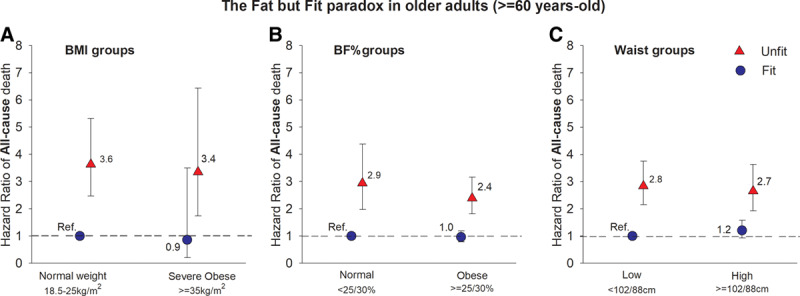
Illustration of the Fat but Fit paradox in older adults. Figure based on the data shown in Table 7 of the article published by Sui et al.^[29]^ Obesity was defined based on BMI using the standard cut-points (BMI ≥ 35 kg/m^2^ to define severe obesity) (A), based on BF% using the cut-point of ≥ 25% and ≥ 30% for men and women, respectively (B), and based on waist circumference ≥ 102 cm and ≥ 88 cm for men and women respectively (C). Unfit-fit was categorized as below-above the age- and sex-specific percentile 20th within each original study. BMI = body mass index; BF% = body fat percent.

Much of what was already mentioned for adults also applies to older adults. The age to define a person as older adult is not universally accepted, being most commonly used the age of 60, 65, or even 70 years. It is well known that fitness, both CRF and muscular strength decline with aging, whereas body fat increases with aging. But how do these 2 important health markers relate to health outcomes and interact during the final years of life? Sui et al.^[29]^ addressed this question using the ACLS data. They followed 2,603 older adults (≥ 60 year-olds) for a mean period of 12 years and examined CRF and fatness as risk factors for all-cause mortality. They observed that CRF predicted the risk of mortality after adjustment for a set of confounders, including adiposity markers such as BMI, BF%, and waist circumference. On the other hand, none of these 3 adiposity markers significantly predicted mortality after additional adjustment for CRF, suggesting that during this period of life, keeping a moderate-to-high fitness level might provide more health benefits and survival than keeping body weight/fat at normal levels. In addition, the authors run combined analyses with fitness and fatness in relation to mortality in this sample of older adults, and the Fat but Fit paradox seems to persist in this last years of life. The normal-weight but unfit individuals had a significantly higher risk of all-cause mortality than their normal-weight fit counterparts, and even a higher risk than obese but fit individuals, supporting the fat but fit paradox (Fig 7). As for younger adults, having moderate-to-high levels of muscular strength has shown to be related to a lower risk of all-cause mortality and cancer mortality in older adults aged 60 years and over.^[28]^ Therefore, fitness assessment in this group seems to be equally important as an indicator of current health status and future prognosis of disease. In addition to the standard incremental treadmill and bike tests conducted in the laboratory, there are available field-based fitness tests and reference values, being the most commonly used the Senior Fitness Test battery proposed by Rikli and Jones: http://www.humankinetics.com/products/all-products/senior-fitness-test-manual-2nd-edition.

Older adults are sometimes considered frail individuals hardly capable to do any real training. However, this has proven to be a wrong assumption. It has been demonstrated that their trainability capacity, for instance to increase their relative strength, is as high as in younger adults. Likewise, they might benefit similarly than their younger fellows from both aerobic and strength training. As an example, we will discuss 2 recent systematic reviews recently conducted in this age group. Bouaziz et al.^[30]^ systematically reviewed the intervention studies testing the effect of aerobic exercise in individuals aged 70 years or older on a diverse number of health outcomes. The authors concluded that there is evidence supporting consistent benefits of aerobic training on cardiovascular, functional, metabolic, cognitive, and quality of life outcomes in older adults, although the exact characteristics of the optimal aerobic program remains unclear.

As in younger adults, muscular strength is also important in older adults, especially if considered the marked decline in muscle mass and risk of sarcopenia and osteoporosis characteristic of aging. Among other benefits, is known that strength training improves functional capacity in older adults and reduces the risk of falls and bone fractures. In this context, Borde et al.^[31]^ systematically reviewed and meta-analyzed the existing randomized controlled trials exploring the effects of strength training (also called resistance training) on muscular strength and muscle morphology in individuals aged 65 years or older. The authors conducted specific dose-response analyses on the 25 studies selected, to draw conclusions about the optimal dose that leads to the best improvements in muscular strength and muscle morphology in terms of volume, intensity, and frequency, among others. Overall, the authors observed a large effect of strength training on improving muscular strength in older adults (ie, effect size = 1.57), whereas the effect was smaller and rated as small-medium (ie, effect size = 0.42) on changes in muscle morphology. Regarding which kind of training leaded to larger improvements in muscular strength, the authors concluded that the optimal dose in older adults seems to be a training period of 50–53 weeks, with an intensity of 70–79% of the 1-RM, a time under tension of 6 s per repetition and a rest of 4 s between repetitions. The review findings also supported to train with a frequency of 2 sessions per week, 2–3 sets per exercise, 7–9 repetitions per set, and a rest between sets of 60 s.

### Summary and future research directions

Fitness and fatness seem to counteract with each other in relation to health outcomes in a similar way in older adults as in younger adults. In fact, the data discussed in this section suggest that a moderately high weight/fat in the later stages of life might be less harmful than in younger ages, while a good fitness level, both CRF and muscular strength, are consistent and independent health markers at these ages. There are data supporting that the Fat but Fit paradox is also present in older adults. Systematic reviews provide strong evidence of multiple health benefits of doing aerobics and muscular strength training in this period of life. Although these reviews have pointed out the type and dose of strength training which is optimal to improve muscular strength, further investigation is needed to clarify the optimal dose and type aerobic exercise leading to best improvements in CRF and other health outcome.

## Conclusions

In this article, we present an overview of what is currently known about fitness and fatness as health markers and how they counteract with each other. A major goal of this article has been to go through the different age groups in a same article, to have an overview of the relevance for health of maintaining fitness, both CRF and muscular strength, at a moderately to high levels, and fatness at low/normal levels, all through the lifespan. We have also briefly covered the relevance of fitness assessment as a vital sign in different age groups, indicating methodological consideration and the availability or not of criterion/reference value allowing a correct interpretation of fitness assessment. Although much remains to be done in this field, an enormous amount of information is nowadays available supporting the importance of this fitness-fatness binomial and should be used in a smart way in public health strategies and for clinical purposes.

## Disclosure

The authors have no financial interest to declare in relation to the content of this article. The Article Processing Charge was paid for by Progress in Preventive Medicine at the discretion of the Editor-in-Chief.
